# Obesity‐Induced Metabolic Priming Exacerbates SARS‐CoV‐2 Inflammation

**DOI:** 10.1111/imm.13934

**Published:** 2025-04-23

**Authors:** Gustavo Gastão Davanzo, Bianca Gazieri Castelucci, Gabriela Fabiano de Souza, Stéfanie Primon Muraro, Larissa Menezes dos Reis, Isabella Bonilha de Oliveira, José Luís Fachi, João Victor Virgilio‐da‐Silva, Marcelo Rodrigues Berçot, Mariane Font Fernandes, Sarah de Oliveira, Nathalia Vitoria Pereira Araujo, Guilherme Ribeiro, Gisele de Castro, Webster Leonardo Guimarães Costa, Adriana Leandra Santoro, Gabriela Flavia Rodrigues‐Luiz, Helison Rafael P. do Carmo, Ikaro Breder, Marcelo A. Mori, Alessandro S. Farias, Daniel Martins‐de‐Souza, Joseph W. Guarnieri, Douglas C. Wallace, Marco Aurélio Ramirez Vinolo, José Luiz Proença‐Módena, Afshin Beheshti, Andrei C. Sposito, Pedro M. Moraes‐Vieira

**Affiliations:** ^1^ Laboratory of Immunometabolism, Department of Genetics, Evolution, Microbiology and Immunology Institute of Biology, University of Campinas São Paulo Brazil; ^2^ Laboratory of Emerging Viruses, Department of Genetics, Evolution, Microbiology and Immunology Institute of Biology, University of Campinas São Paulo Brazil; ^3^ Department of Clinical Medicine, School of Medical Sciences University of Campinas São Paulo Brazil; ^4^ Laboratory of Immunoinflammation, Department of Genetics, Evolution, Microbiology, and Immunology Institute of Biology, University of Campinas São Paulo Brazil; ^5^ Laboratory of Neuroproteomics, Department of Biochemistry and Tissue Biology Institute of Biology, University of Campinas (UNICAMP) São Paulo Brazil; ^6^ Obesity and Comorbidities Research Center (OCRC) University of Campinas São Paulo Brazil; ^7^ Experimental Medicine Research Cluster (EMRC) University of Campinas São Paulo Brazil; ^8^ Department of Biochemistry and Tissue Biology Institute of Biology, University of Campinas (UNICAMP) São Paulo Brazil; ^9^ Autoimmune Research Laboratory, Department of Genetics, Microbiology, and Immunology Institute of Biology, University of Campinas (UNICAMP) São Paulo Brazil; ^10^ Center for Mitochondrial and Epigenomic Medicine, Division of Human Genetics The Children's Hospital of Philadelphia Philadelphia Pennsylvania USA; ^11^ Broad Institute of MIT and Harvard Cambridge Massachusetts USA; ^12^ Center for Space Biomedicine, McGowan Institute for Regenerative Medicine, Department of Surgery University of Pittsburgh Pittsburgh Pennsylvania USA

**Keywords:** COVID‐19, inflammation, innate immunity, monocyte, obesity

## Abstract

Despite the early recognition that individuals living with obesity are more prone to develop adverse outcomes during COVID‐19, the mechanisms underlying these conditions are still unclear. During obesity, an accumulation of free fatty acids (FFAs) in the circulation promotes low‐grade inflammation. Here, we show that FFAs induce epigenetic reprogramming of monocytes, exacerbating their inflammatory profile after SARS‐CoV‐2 infection, a mechanism named metabolic‐primed immunity. Monocytes from people with obesity or primed with palmitate, a central component of circulating FFAs, presented elevated viral load and higher gene expression of IL‐6. Palmitate‐primed monocytes upregulate fatty acid oxidation and FFAs entry into the mitochondria. FFA‐derived acetyl‐CoA is then converted into citrate, exiting the mitochondria and is used to support H3K18 histone acetylation, which regulates IL‐6 accessibility. Ingestion of palm oil by lean and healthy individuals increased circulating FFAs levels and was sufficient to exacerbate the inflammatory profile of monocytes upon SARS‐CoV‐2 infection. Our findings demonstrate that obesity‐derived FFAs induce the metabolic priming of monocytes, which exacerbates the inflammatory response observed in people with severe COVID‐19.

## Graphical abstract

Monocytes primed under obesogenic conditions, such as increased FFA levels (i.e., palmitate) exhibit exacerbated gene expression of inflammatory cytokines. The increased proinflammatory response of palmitate‐primed and SARS‐CoV‐2‐infected monocytes is not dependent on glycolytic metabolism but instead on the entry of FFAs into the mitochondria, followed by the export of citrate from the mitochondria to the cytosol. The accumulation of cytosolic citrate increases histone acetylation and the expression of pro‐inflammatory cytokines, such as IL‐6. Increased pro‐inflammatory levels are associated with the cytokine storm, commonly associated with severe COVID‐19 disease.

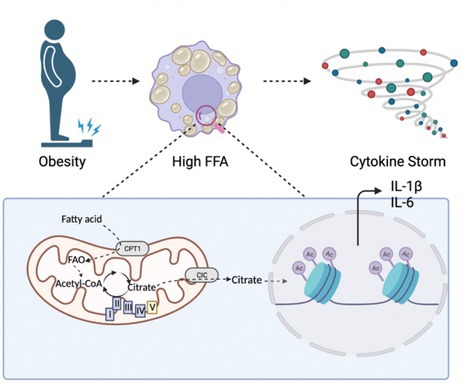

## 1 Introduction

The coronavirus disease 2019 (COVID‐19) is characterised by high morbidity and mortality rates in populations with comorbidities, including people with obesity, cardiovascular disease, hypertension, advanced age and type 2 diabetes mellitus (T2D) [1, 2, 3, 4]. Obesity is a complex and multifactorial condition and a worldwide public health concern. In individuals with obesity, the imbalance between energy intake and expenditure leads to elevated circulating FFA levels. These elevated FFAs levels increase the expression of proinflammatory mediators in the serum, such as TNF‐α, IL‐6 and IL‐1β [5, 6].

Population‐level studies have reported a higher risk of complications and death in people with obesity infected with COVID‐19 [7, 8, 9]. Obesity also increases the risk for T2D, and both are considered risk factors for severe COVID‐19 disease [10]. Epidemiological data show that not all people living with obesity have T2D [11], suggesting that the mechanisms influencing COVID‐19 severity in these conditions are distinct. Despite the early recognition that individuals with obesity are more prone to adverse outcomes during COVID‐19, the mechanisms underlying these conditions, especially in individuals who do not have diabetes, remain unclear.

Given the established link between obesity and adverse COVID‐19 outcomes, understanding the underlying immune mechanisms, mainly how metabolic factors like FFAs might drive a heightened immune response, is crucial. This led us to explore the concept of primed immunity [12], wherein immune cells undergo metabolic reprogramming, resulting in an altered response to a second, unrelated challenge. Different metabolic substrates can be used by immune cells to modify their epigenetic landscape. Lipid‐derived acetyl‐CoA is a primary source of acetyl groups for histone acetylation in various cell types [12]. Given that SARS‐CoV‐2 infects and induces an inflammatory profile in different immune cells, including dendritic cells, monocytes and macrophages [13, 14, 15, 16], which directly impact disease severity, we hypothesise that elevated levels of FFAs in the circulation of people with obesity may prime monocytes and macrophages. Upon a second challenge, such as SARS‐CoV‐2 infection, this priming leads to the exacerbated production of several pro‐inflammatory cytokines, including IL‐6, TNF and IL‐1β, in a process known as a cytokine storm, which is commonly found in people with severe COVID‐19 [17].

Here, we show that monocytes primed under obesogenic conditions, such as increased FFA levels (i.e., palmitate), are more susceptible to SARS‐CoV‐2 infection and exhibit exacerbated gene expression of inflammatory cytokines. The increased proinflammatory response of palmitate‐primed and SARS‐CoV‐2‐infected monocytes is not dependent on glycolytic metabolism but instead on the entry of FFAs into the mitochondria, followed by the export of citrate from the mitochondria to the cytosol. The accumulation of cytosolic citrate increases the acetylation of a specific histone residue (H3K18), which was previously associated with elevated IL‐6 levels in individuals with obesity. We found that blood samples of individuals living with obesity or palmitate priming enhanced the acetylation of H3K18 and is associated with increased gene expression of this IL‐6 and pro‐IL‐1β. Therefore, obesity‐derived FFAs induce a distinct type of primed immunity, called ‘metabolic primed immunity,’ leading to heightened gene expression of inflammatory cytokines during SARS‐CoV‐2 infection. Finally, we demonstrate that the elevation of FFAs in lean and healthy volunteers induces metabolic primed immunity in circulating monocytes, which is sufficient to enhance the inflammatory profile of these cells after SARS‐CoV‐2 infection.

## 2 Methods

### 2.1 Sample Acquisition From Lean/Overweight and Individuals Living With Obesity

The study was approved by the Brazilian Committee for Ethics in Human Studies (CAEE: 36590020.1.0000.5404). Formal consent was obtained from all individuals. Nine lean/overweight adiposity < 16.2% and five individuals living with obesity over 18 years of age, without diabetes mellitus, with a BMI greater than 30 kg/m^2^, adiposity > 25% and with hypertriglyceridemia, unvaccinated against SARS‐CoV2, were recruited at the Clinical Research Centre of the Faculty of Medical Sciences at UNICAMP. Individuals living with obesity (three males and two females, aged between 41 and 65 years old). Blood collection (20 mL in EDTA) was performed on the day of the clinical evaluation. The samples used in this manuscript were obtained in 2020 from SARS‐CoV‐2 unvaccinated individuals.

### 2.2 PBMCs Isolation

Peripheral blood from healthy donors was obtained from buffy coats provided by the Haematology and Hemotherapy Centre of the University of Campinas (SP‐Campinas, Brazil). The study was approved by the Brazilian Committee for Ethics in Human Studies (CAEE: 31622420.0.0000.5404). Peripheral blood mononuclear cells (PBMCs) were isolated by density‐gradient centrifugation using 1.077 g × mL^−1^ Ficoll gradient. Briefly, buffy coats were gently mixed and then diluted (1:1) with phosphate buffer saline (PBS) and carefully transferred to a 50‐mL tube containing 10 mL of Ficoll, which was centrifuged at 2700 rpm for 20 min at room temperature. The PBMC monolayer was transferred to a new tube and washed with PBS. Total PBMCs were cultured as adherent monolayers (1.5 × 10^6^ cell × mL^−1^) in RPMI 1640 (serum‐free) with 1% Penicillin–Streptomycin at 37°C with a 5% CO_2_ atmosphere. After 2–3 h of adhesion, cells were washed with PBS and incubated with palmitate or BSA activation media in RPMI 1640 containing 10% fetal bovine serum (FBS) and 1% Penicillin–Streptomycin at 37°C with a 5% CO_2_ atmosphere. All the experiments were performed before COVID‐19 vaccines. The list of reagents, including antibodies, can be found in Supporting Information: Table 3.

### 2.3 Palmitate Preparation

A 5% bovine serum albumin fatty acid (FA)‐free (Sigma Aldrich—A7030) was made in 1× PBS and dissolved under agitation. A 20 mM solution of sodium palmitate (Sigma Aldrich—P9767) was prepared using miliQ water in a 15 mL tube. The solution was dissolved in boiled water under agitation for 20 min. One volume of 20 mM palmitate solution was mixed with three volumes of BSA solution to get a 5 mM palmitate solution. The clear solution was filtered and added to RPMI to achieve a 0.2 mM palmitate activation medium. The BSA controls were made with a 5% solution dissolved in RPMI. The list of reagents, including antibodies, can be found in Supporting Information: Table 3.

### 2.4 Reagents and Infection

PBMCs were treated with inhibitors 18 h before viral infection in RPMI‐containing palmitate or BSA controls. Cells were infected with SARS‐CoV‐2 at MOI 0.1 under continuous agitation at 15 rpm for 1 h for virus absorption. MOI of 0.1 was used after titration with MOIs of 0.1, 1 and 3 as described [18]. After infection, cells were washed with prewarmed PBS twice and incubated in RPMI containing 10% FBS and 1% Penicillin–Streptomycin for 24 h at 37°C with a 5% CO_2_ atmosphere.

### 2.5 Viruses and Cell Lines

The SARS‐CoV‐2 B original lineage (GenBank HIAE‐02 SARS‐CoV‐2/SP02/human/2020/BRA accession number MT126808.1), variant Omicron (HIAE W.A. EPI_ISL_6901961) and variant Delta (B.1.617.2‐like) (GISAID: EPI_ISL_3461104) were isolated in Brazil and kindly donated by Professors Dr. Edson Luiz Durigon, Dr. Ester Sabino (IMT‐SP), Dr. Fernando Spilki (FEEVALE‐SC) and Dr. João Renato Rebello Pinho (HIAE). All Sars‐CoV‐2 virus stocks were propagated in Vero cells (CCL‐81; ATCC, Manassas, VA, USA), and the supernatant was harvested at 2–3 dpi. Plaque assays determined the viral titers on Vero cells. Vero CCL‐81 cells were cultivated in DMEM supplemented with 10% heat‐inactivated FBS and 1% Penicillin–Streptomycin and incubated at 37°C with a 5% CO_2_ atmosphere.

### 2.6 RNA Extraction, Viral Load and Gene Expression Analyses

After each experimental protocol, cells were washed with 1 × PBS, and 200 uL of TRIzol Reagent (Thermo Scientific, code:15596026) was added to the plate. Cells were scraped and transferred to a new 1.5 mL sterile tube. Total RNA extractions were performed using TRIzol Reagent according to the manufacturer's instructions. RNA concentration was determined using the NanoDrop 2000 spectrophotometer (Thermo Scientific). According to the manufacturer's instructions, the total RNA extracted was reverse transcribed using a Reverse Transcriptase cDNA (Thermo Scientific, code 4311235) synthesis kit. Specific SARS‐CoV‐2 N1 primers targeting the N1 region were used as previously described for viral load detection [18]. Viral load and gene expression qRT‐PCR were performed using QuantiNova SYBR Green PCR kit (Qiagen, code:208056). All qRT‐PCR reactions were performed using the BIO‐RAD CFX394 Touch Real‐Time PCR Detection System on 384‐well plates. Gene expression fold change was calculated with the ΔΔ*C*_t_ method. The list of reagents can be found in Supporting Information: Table 3. The sequences of the primers used are available in Supporting Information: Table 4.

### 2.7 Real‐Time Metabolic Assays

Using the XFe24 plate (Seahorse Bioscience), 4 × 10^6^ PBMCs were plated and activated as described above. At the end of the experiment, cells were starved according to the Glycolytic Stress, Mito Stress or Mito Fuel Tests following the manufacturer's protocols. All results were normalised to protein content.

### 2.8 Palm Oil Ingestion Protocol

The study was approved by the Brazilian Committee for Ethics in Human Studies (CAEE: 36590020.1.3001.5474). One week before the experiment, the research participants (*n* = 4, unvaccinated against SARS‐CoV 2) received nutritional guidance to restrict the consumption of foods rich in palmitic acid in preparation for the test diet. The restriction was maintained for 7 days before the intervention with the test diet. During the preparation period for the test diet, the participants maintained their usual work routine.

The test diet consisted of Crude Palm Oil extracted from the pulp of the oil palm fruit *Elaeis guineensis* . The test diet was administered in two phases: the first dose (70 mL) replaced breakfast, and the second (70 mL) replaced dinner. The volunteers fasted before consuming the test diet for breakfast and should not eat other foods for lunch. After administering the test diet, the restriction on the consumption of palmitic acid was maintained for another 24 h. To ensure adequate energy and nutritional intake on the day of the intervention, research participants were offered a snack in the morning and another in the afternoon consisting of foods that did not interfere with lipemia—100 g of banana and 100 g of apple. The Nutritional Assessment and Dietary Prescription Software—Dietbox was used to calculate the snacks. A blood collection (20 mL in EDTA) was performed before administration of the test diet to measure circulating FA by HPLC‐MS. After administering the test diet, blood samples were taken (20 mL in EDTA) at 4, 12 and 24 h to evaluate the FA profile by HPLC‐MS. At all‐time points, an additional 20 mL of blood was collected to separate the participants' monocytes. Participants were located at the Clinical Research Centre for blood collection during the first 12 h. Twenty‐four hours after the first ingestion, the volunteers returned to the Clinical Research Center for blood collection. A last blood collection was performed after 7 days of the test diet.

### 2.9 Histone Acylation by FACS

After the cell culture infection, monocytes were washed with sterile PBS and scraped with 200 μL of cold PBS. Cell viability (BD Biosciences—V510, code 564406) and CD14 (BD Biosciences—APC‐Cy7, code: 555397) cell surface staining were diluted in FACS buffer at 4°C for 20 min in the dark after the blockade of Fc receptors with purified anti‐CD16/CD32 (Biolegend). Cells were stained for viability and then fixed for intracellular acetylated lysine staining using the eBioscience Transcription Factor staining Kit for cytoplasmic or nuclear proteins, according to the manufacturer's instructions. First, we incubated the fixed cells for 2 h with rabbit monoclonal antibodies to histone H3 acetyl K18 (anti‐H3K18ac; Abcam, code: ab1191) or histone H4 acetyl K8 (anti‐H4K8ac; Abcam, code: ab15823). Next, after washing the samples, we added the Alexa Fluor‐647 donkey polyclonal secondary antibody to rabbit IgG (Abcam, code: A‐31573) for 1 h. Samples were washed and then acquired on BD FACS A5 (BD Biosciences) using BD FACS Software (BD biosciences), and FACS data were analysed using FlowJo v.9.5.2 software (Tree Star). The gate strategy is shown in Supporting Information: Figure 4C.

### 2.10 Mitochondrial Probes

PBMCs were collected 24 h after infection and subjected to staining with BD Horizon Fixable Viability Stain 510 (BD Biosciences, code: BDB564406), MitoSOX Red Mitochondrial ROS Indicator (Thermo Science, code: M36008), Mito Tracker Deep Red (Thermo Science, code: M46753) and anti‐CD14‐PE‐Cy7 (Biolegend, code: 325617) at 37°C for 15 min. The cells were washed with PBS, fixed with 4% p‐formaldehyde for 30 min at 4°C, and then moved to polypropylene FACS tubes. A FACS Symphony (BD Biosciences, San Diego, CA, USA) flow cytometer was employed to analyse the cells, and FlowJo software was utilised to evaluate the data. The gate strategy is shown in Supporting Information: Figure 4C.

### 2.11 RNA‐Sequencing (RNAseq)

PBMCs were activated with palmitate or BSA for 18 h and infected with Mock control or SARS‐CoV‐2 as described above. At the end of the protocol, cells were washed with PBS, and RNA was extracted using the RNAeasy mini kit according to the manufacturing instructions (Qiagen, code: 4004). RNA integrity was analysed on a Bioanalyzer RNA Pico 6000 chip at the Core Facility for Scientific Research—University of São Paulo (CEFAP‐USP). cDNA library construction and sequencing were performed by BGI Global Genomics Services using the DNBSeq platform. Illumina sequencing adapters and low‐quality reads were removed with Trimmomatic. Trimmed reads were aligned to the human reference genome (GRCh38) by STAR. Aligned reads were mapped to features using HTSeq, and differential expression analyses were performed using the DESeq2 package. Genes under 5 TPM in at least three samples were excluded before statistical analysis. DEGs were selected using the adjusted *p* < 0.05 and log2 fold change (LFC) > 0.5 as cutoffs. Differential gene expression (DGE) data were utilised to find key mitochondrial and immune‐related gene regulation. The customised gene lists we curated are available [19]. Heatmaps were displayed using pheatmap (1.0.12) in R for all genes, and the significant genes were designated by a * for adjusted *p* value or FDR < 0.05.

### 2.12 Gene Set Enrichment Analysis (GSEA)

We utilised fast Gene Set Enrichment Analysis (fGSEA) for pathway analysis. Pathway analysis was done utilising both MsigDB gene sets [20, 21] (i.e., specifically Hallmark and KEGG) and custom‐made Gene Set files from either MitoPathway or the genes we curated available [19]. Using fGSEA, all samples were compared to controls, and the t‐score statistics defined the ranked list of genes. One thousand permutations of the genesets determined the statistical significance [22], and lollipop plots were made using ggplot2 (ver 3.3.5). Sequencing data are deposited with the project number PRJNA951408.

### 2.13 ChIP‐qPCR

Monocytes were cross‐linked with PBS‐1% formaldehyde for 10 min at room temperature in gentle shaking, quenched with glycine (0.125 M final concentration), washed with ice‐cold PBS and frozen at −80°C until the moment of chromatin immunoprecipitation (ChIP), adapted from the conventional protocol [23]. Briefly, the cells were thawed on ice and resuspended with lysis buffer (50 mM Tris–HCl pH 7.5, 10 mM EDTA, 1% SDS, 1 mM PMSF, 1× Protease Inhibitor Cocktail, 5 mM Butyrate) for 10 min. The chromatin was sheared to a 100–500 bp fragment size range by a water‐cooled Bioruptor Sonicator (Diagenode) for 20 cycles of 30 s ON, 30 s OFF and high power at 4°C. The lysates were cleared by centrifugation at 20 800*g*, 4°C for 10 min, and sonication efficiency was assessed by running 10% of each sample on a 2% agarose gel. After confirming the fragment size, the final volume of the supernatant was completed to 800 μL with RIPA ChIP buffer (RIPA low salt buffer, 1 mM PMSF, 1× protease inhibitor cocktail, 5 mM butyrate) and the same amount of chromatin for each sample was used for ChIP, with 2% of the volume reserved for the Input. At the same time, Protein A and G Dynabeads (Invitrogen, code: 10001D and 10003D) were washed with RIPA Low Salt buffer (10 mM Tris–HCl pH 7.5, 140 mM NaCl, 1 mM EDTA, 0.5 mM EGTA, 1% Triton X‐100, 0.1% SDS, 0.1% Na‐deoxycholate) using a magnetic stand and incubated with 5 μg of the Anti‐H3K18ac antibody (Abcam, code: ab1191) for 4 h in slow rotation at 4°C. The beads conjugated to antibodies were rewashed with RIPA low salt and resuspended in the respective sample supernatant for the immunoprecipitation on a tube rotator with slow rotation at 4°C overnight. The complexes formed were washed with ice‐cold RIPA Low Salt buffer and TE buffer (10 mM Tris–HCl, pH 8.0, 1 mM EDTA). The reverse‐crosslinking was performed in both chromatin and input samples through incubations with Elution buffer (20 mM Tris–HCl pH 7.5, 5 mM EDTA, 50 NaCl) for 20 min at 65°C at 1300 rpm, followed by 30 min at 37°C with the addition of RNase A solution (Invitrogen, code: 12091021) and 1% SDS to remove contaminating RNA, and 2 h at 65°C with the addition of Proteinase K (Ambion, code: AM2548) also to digest proteins. The samples were then centrifuged for 1 min at 11 600*g* at room temperature, purified with the minute PCR purification kit (QIAGEN, code: 28004), eluted in 22 μL autoclaved Milli‐Q water, and frozen at −20°C until the moment of the qPCR, using antibodies listed in Supporting Information: Table 3 and primers in Supporting Information: Table 4.

### 2.14 Statistical Analysis

RNA‐seq from three samples of each group was analysed as described above. Additional data were plotted and analysed using the GraphPad Prism 8.0 software (GraphPad Software, San Diego, CA). For analyses between the two groups, the student's *t* test was used. For comparisons among more than two groups, either One‐way or Two‐way ANOVA, followed by Tukey post hoc tests, was used. Differences were considered statistically significant when *p* < 0.05. In vitro data represent mean ± SEM of at least three independent experiments (three biological samples) performed in triplicate (three technical replicates).

## 3 Results

### 3.1 Obesity‐Primed Monocytes Infected With SARS‐CoV‐2 Display Exacerbated Inflammatory Profiles

**FIGURE 1:**
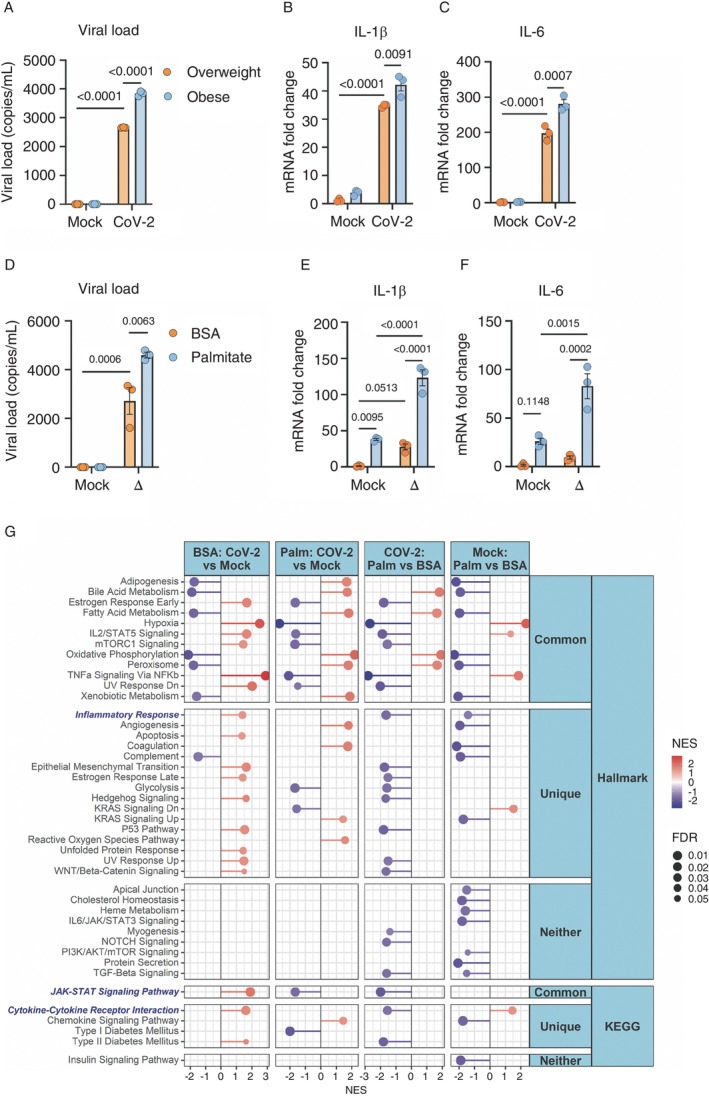
Blood from people living with obesity or palmitate‐primed monocytes presents worsening in SARS‐CoV‐2 infection and exacerbated inflammatory profile. (A–C) Monocytes derived‐PBMCs from healthy lean/overweight or individuals with obesity (details in Supporting Information: Table 1) were infected with CoV‐2 (SARS‐CoV‐2, variant B1) for 1 h. Cells were washed, and the viral load and cytokines were measured by qPCR 24 h later. Each group consists of *n* = 3 samples/group. Data represents mean ± SEM of at least three independent experiments *n* = 3 samples/group; two‐way ANOVA with Tukey's post hoc tests. (D–F) Monocytes from healthy buffy coats primed with BSA/palmitate (0.2 mM) and infected with mock or the delta variant of SARS‐CoV‐2 (∆). Viral load and cytokines gene expression were measured by qPCR 24 h after the infection. Each group consists of *n* = 3 samples/group. Data represents mean ± SEM of at least three independent experiments *n* = 3 samples/group; two‐way ANOVA with Tukey's post hoc tests. (G) Lollipop plots were constructed to visualise statistically significant (FDR < 0.05) up/down‐regulated pathways related to the immune system, as determined from the KEGG and Hallmarks databases using Gene Set Enrichment Analysis (GSEA) on RNA‐seq data from monocytes sourced from healthy buffy coats. These monocytes were primed with BSA/palmitate and infected with either mock or CoV‐2 (SARS‐CoV‐2, B1 variant). The normalised enrichment score (NES) indicates the relative degree of change in gene sets and is adjusted for gene set size. In the plots, red intensity represents upregulated pathways, blue intensity represents downregulated pathways and specific immune system and inflammation‐related processes or pathways are highlighted in blue. Pathways are categorised as ‘Common’ if they are significantly regulated in both BSA and Palmitate CoV‐2 versus Mock conditions, ‘Unique’ if they are only considerably regulated in either BSA or Palmitate CoV‐2 versus Mock conditions and ‘Neither’ if they are not significantly regulated in either BSA or Palmitate CoV‐2 versus Mock conditions.

While it is well established that obesity is a risk factor for severe COVID‐19, and a link between monocyte/macrophage‐driven inflammation and severe disease has been identified, the exact mechanisms contributing to the increased severity of COVID‐19 in individuals with obesity remain unclear [13]. Previous research from our group demonstrated that monocytes isolated from diabetic and insulin‐resistant individuals infected with SARS‐CoV‐2 presented an increased viral load and higher expression of proinflammatory cytokines [18]. Additionally, we showed that elevated glucose levels favour SARS‐CoV‐2 infection and proinflammatory cytokine expression, driven by glycolysis [18]. To distinguish the effects of obesity from those of insulin resistance on the inflammatory profile of monocytes, we analysed monocytes isolated from two clinical groups: lean/overweight insulin‐sensitive (BMI 26 ± 3 Kg/m^2^, HOMA‐IR 2.5 ± 1.8, insulin 10 ± 7 mUI/mL, HbA1c 5.0% ± 0.3%) and individuals with obesity and insulin‐sensitive (BMI 33 ± 3 Kg/m^2^, HOMA‐IR 2.6 ± 1.6, insulin 11 ± 6 mUI/mL, HbA1c 5.5% ± 0.3%) (Supporting Information: Table 1). Monocytes from individuals living with obesity exhibited an increased viral load and elevated gene expression of IL‐6 and pro‐IL‐1β compared to those from lean/overweight individuals (Figure 1A–C and Supporting Information: Figure 1A). This suggests that in addition to elevated glucose levels seen in diabetic individuals, other factors contribute to the exacerbated inflammatory profile in SARS‐CoV‐2‐infected monocytes from individuals living with obesity.

Elevated levels of FFAs, such as palmitate, trigger a proinflammatory profile in macrophages [24, 25]. Since obese individuals have increased circulating FFA levels, we treated monocytes from healthy donors with palmitate (in the presence of normal glucose levels, i.e., 5.5 mM) to mimic a nondiabetic obesogenic environment. Monocyte activation with palmitate for 18 h before infection (referred to as priming) with different SARS‐CoV‐2 variants (B1, Delta, Omicron) led to an increased viral load and elevated gene expression of IL‐6 and IL‐1β in all variants tested (Figure 1D–F and Supporting Information: Figure 1B–H). These results indicate that monocyte activation under obesogenic conditions, even in the absence of elevated glucose levels, is sufficient to enhance SARS‐CoV‐2 infection and drive the gene expression of proinflammatory cytokines.

To further explore the biological pathways and mechanisms associated with the exacerbated inflammatory profile of obesity‐primed monocytes under SARS‐CoV‐2 infection, we performed bulk RNA‐seq of monocytes primed with BSA or palmitate for 18 h, followed by SARS‐CoV‐2 infection. SARS‐CoV‐2 infection (see column BSA: CoV‐2 vs. Mock control) induces upregulation of various inflammatory pathways, such as those involved in the inflammatory response, interferon‐alpha response, interferon‐gamma response, TNF‐alpha signalling via NF‐κB, cytokine‐cytokine receptor interaction, the JAK–STAT signalling pathway and the NOTCH signalling pathway (Figure 1G, Supporting Information: Figure 1I,J). Several genes associated with innate immune inflammation (JUN, TNF, TNFAIP6, TNFSF9, TNFSF15, TNFRSF9, NFKB1, IKBK3 and IL‐1A), cytokines and interleukins (CSF3, IL‐1B, IL‐6, IL‐10 and IL‐21R), as well as genes involved in different types of cell death (CASP1, IL‐1R1, FAS, TNFRSF0B, TNF, BCL2L11, BBC3, PMAIP1, XIAP, BCL2 and BCL2L1), were upregulated upon SARS‐CoV‐2 infection (Supporting Information: Figure 2A–D). It is essential to highlight that several groups have demonstrated inflammatory and metabolic modifications induced by SARS‐CoV‐2 infection at the protein level, including our research [18, 26, 27]. These data suggest that SARS‐CoV‐2 infection in monocytes induces robust inflammatory activation involving elevated gene expression of several cell death pathways, including the inflammasome. However, further functional validation is necessary to confirm these pathways' involvement and mechanistic contribution.

The activation of monocytes with palmitate, in the absence of a virus (see the column Mock: Palm × BSA), is sufficient to induce a low‐grade inflammatory response with upregulation of TNF alpha signalling via NF‐κB, cytokine‐cytokine receptor interaction and the JAK–STAT signalling pathway (Figure 1G; Supporting Information: Figure 1I,J), as well as genes involved in inflammation (JUN, TNFRSF9) and cytokines and interleukins (CSF3, IL2RB, IL23R) (Supporting Information: Figure 2A–D).

Priming monocytes with palmitate altered the proinflammatory profile induced by SARS‐CoV‐2 infection (Palm _ CoV‐2) (Figure 1G; Supporting Information: Figure 1H,I). Palmitate priming also had profound effects on the metabolic pathways activated by SARS‐CoV‐2 or by treatment with palmitate alone, such as decreased expression of genes associated with glycolysis, mTORC1 signalling and hypoxia and upregulation of pathways related to FA metabolism, citrate TCA cycle, pyruvate metabolism and oxidative phosphorylation (OXPHOS) (Figure 1G; Supporting Information: Figure 1I,J). However, the metabolic adaptation induced by palmitate priming did not further amplify the changes triggered by SARS‐CoV‐2 infection or palmitate alone, suggesting a convergence in metabolic rewiring. Therefore, priming monocytes with palmitate affected the inflammatory and metabolic signatures of SARS‐CoV‐2‐infected monocytes.

### 3.2 Palmitate Priming of Monocytes Inhibits SARS‐CoV‐2 Or Palmitate‐Induced Glycolysis

**FIGURE 2:**
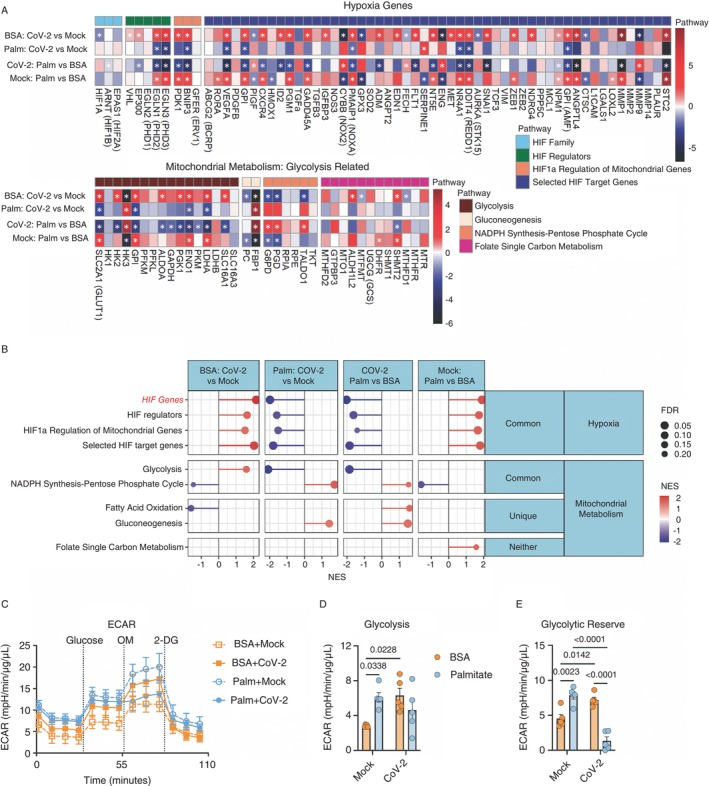
Palmitate priming of monocytes abrogates palmitate or SARS‐CoV‐2‐induced glycolysis. (A) Heatmaps of Log_2_ fold‐change values for glycolysis and hypoxia pathways. The colour bar represents the Log_2_ fold change values and the * indicated genes with adj. *p* value (FDR) < 0.05.(*n* = 3/group). (B) Lollipop plots for statistically significant (FDR < 0.25) up/down‐regulated pathways for custom mitochondrial‐related pathways determined by Gene Set Enrichment Analysis (GSEA) from the RNA‐seq on monocytes from healthy buffy coats primed with BSA/palmitate (0.2 mM) and infected with mock or CoV‐2 (SARS‐CoV‐2, B1 variant). The normalised enrichment score (NES) represents the relative degree to which a gene set is changed and is corrected for gene set size. The red intensity in the plot indicates the degree of upregulated pathways, and the blue intensity indicates the degree of downregulated pathways. The pathways are organised by the following: ‘Common’ = pathways are significantly regulated for both BSA and Palm CoV‐2 versus Mock, ‘Unique’ = pathways are only significantly regulated for either BSA and Palm CoV‐2 versus Mock, and ‘Neither’ = pathways are not significantly regulated for either BSA or Palm CoV‐2 versus Mock (*n* = 3/group). (C) Monocytes from healthy buffy coats primed with BSA/palmitate for 24 h and infected with mock or CoV‐2 (SARS‐CoV‐2, B1 variant) for 1 h, were washed, and 24 h later submitted to a Glyco Stress test using Seahorse. Glucose (10 mM); OM (1 μM)—oligomycin; 2‐DG (50 mM)—2‐deoxy‐glucose. (D) Glycolysis was calculated by the difference in the maximum rate measurement before oligomycin and the last measurement before glucose. (E) Glycolytic reserve was calculated by the difference between glycolytic capacity (maximum measurement after oligomycin—last measurement before glucose injection) and glycolysis. Data represents mean ± SEM of at least three independent experiments performed in triplicate for each sample; two‐way ANOVA with Tukey's post hoc tests. *n* = 5 samples/group.

Next, we focused on the major metabolic pathways involved in the metabolic priming of monocytes. Palmitate treatment or SARS‐CoV‐2 infection upregulates the glycolytic pathway in an HIF‐1α‐dependent manner [18, 28, 29]. Our transcriptomic data indicate that monocytes treated with palmitate or infected with SARS‐CoV‐2 display increased gene expression of HIF‐1α‐target genes, including those involved in angiogenesis (ANGPTL4, FLT1, VEGFA, EDN1), apoptosis (PMAIP1), DNA damage pathways (GADD45A), regulation of mitochondria (BNIP3 and PDK1) and the glycolytic pathway (SLC2A1, HK2, GPI, PGK1, ENO1 and LDHA) (Figure 2A,B). Real‐time measurements of the extracellular acidification rate (ECAR), indicative of aerobic glycolysis, confirmed that priming monocytes with palmitate or infection with SARS‐CoV‐2 alone induced higher glycolysis and glycolytic reserve in monocytes (Figure 2C–E). However, Palm+CoV‐2 displayed an opposite profile for glycolytic‐ and HIF‐1α‐target genes compared to palmitate‐treated or SARS‐CoV‐2‐infected monocytes (Figure 2A,B), in agreement with our RNAseq data. Palm+CoV‐2 also showed reduced glycolytic reserve (Figure 2E). Our data suggest that palmitate priming abrogates SARS‐CoV‐2‐induced glycolysis.

### 3.3 Palmitate‐Priming Favours Fatty Acid Oxidation in Monocytes Infected With SARS‐CoV‐2

**FIGURE 3:**
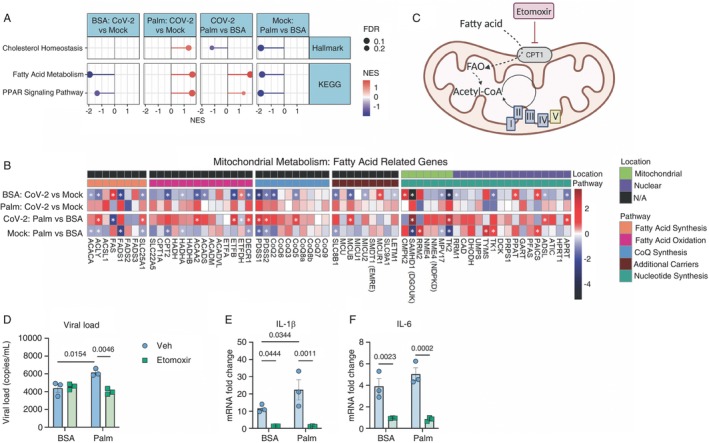
Palmitate‐priming induction of fatty acid oxidation SARS‐CoV‐2 infected monocytes is associated with an exacerbated viral load and inflammatory‐cytokine gene expression. (A) Lollipop plots for statistically significant (FDR < 0.25) up/down‐regulated pathways for fatty acid‐related pathways/processes determined from the KEGG and Hallmarks databases by Gene Set Enrichment Analysis (GSEA) from the RNA‐seq on monocytes from healthy buffy coats primed with BSA/palmitate (0.2 mM) and infected with mock or CoV‐2 (SARS‐CoV‐2, B1 variant). The normalised enrichment score (NES) represents the relative degree to which a gene set is changed and is corrected for gene set size. The red intensity in the plot indicates the degree of upregulated pathways, and the blue intensity indicates the degree of downregulated pathways. Specific immune system and inflammation‐related processes or pathways are highlighted in blue. (*n* = 3/group). (B) Heatmaps of Log2 fold‐change values for fatty acid metabolism‐related pathways from the RNA‐seq on monocytes from healthy buff coats primed with BSA/palmitate (0.2 mM) and infected with mock or CoV‐2 (SARS‐CoV‐2, B1 variant). The colour bar represents the Log2 fold change values and the * indicated genes with adj. *p* value (FDR) < 0.05. (*N* = 3/group). (C) Schematic representation of β‐oxidation of fatty acids contributing to the TCA cycle, ETC. (D–F) Monocytes from healthy buffy coats were pretreated with 3 μM etomoxir for 1 h, primed with BSA/palmitate (0.2 mM) for 18 h, and infected with CoV‐2 (SARS‐CoV‐2, B1 variant) for 1 h. Cells were washed, and viral load and cytokines were measured by qPCR 24 h later. Data represents mean ± SEM of at least three independent experiments performed in triplicate for each sample (*n* = 3 samples). Two‐way ANOVA with Tukey's post hoc tests.

The breakdown of FFAs by fatty acid oxidation (FAO) generates acetyl‐CoA units, which can become one of the primary energy sources in the cell [30]. The entry of FFAs into the mitochondria requires the carnitine palmitoyl transferase (Cpt1) system. Cpt1 is the first component and rate‐limiting step in the transport of FFAs into the mitochondrial intermembrane space, which converts fatty‐acyl CoA into acylcarnitine. A translocase system then shuttles the acylcarnitine to the mitochondrial matrix, oxidising it into acetyl‐CoA molecules that enter the TCA cycle [31]. Palm+CoV‐2 upregulated the carnitine shuttle, carnitine synthesis and transport, lipid metabolism, cholesterol homeostasis, FA metabolism and PPAR pathway (Figure 3A; Supporting Information: Figure 3A). The elevation of genes involved in FAO (ACAA2, ETFB and DECR1) (Figure 3B) suggests that FAO is degrading palmitate. Thus, we inhibited Cpt1 with low doses of etomoxir (Figure 3C). Inhibition of Cpt1 in Palm+CoV‐2 monocytes decreased the viral load and the gene expression of inflammatory cytokines (Figure 3D–F). Together, these data suggest that FAO‐derived carbons affect viral replication and the inflammatory response of monocytes.

### 3.4 Palmitate Priming Induces Mitochondrial Dysfunction in SARS‐CoV‐2‐Infected Monocytes

Acetyl‐CoA derived from FAO enters the TCA cycle and can be oxidised to supply electrons to the electron transport chain (ETC) for ATP production. We tested the fuel oxidation of SARS‐CoV‐2‐infected monocytes to glucose, glutamine and FFAs, the three main carbon sources for mitochondrial respiration. Both glucose and glutamine are known to be important carbon sources for ATP production in SARS‐CoV‐2‐infected cells, but the contribution of FFAs to ATP production is unknown. We observed that, as expected, both glucose and glutamine are important fuel sources in SARS‐CoV‐2‐infected monocytes (Supporting Information: Figure 3B). In contrast, FFAs were not used as a fuel source in SARS‐CoV‐2‐infected monocytes (Supporting Information: Figure 3B). Our data suggest that palmitate‐derived carbons are not oxidised to produce ATP, and these carbons must have an alternative fate in SARS‐CoV‐2‐infected monocytes.

**FIGURE 4:**
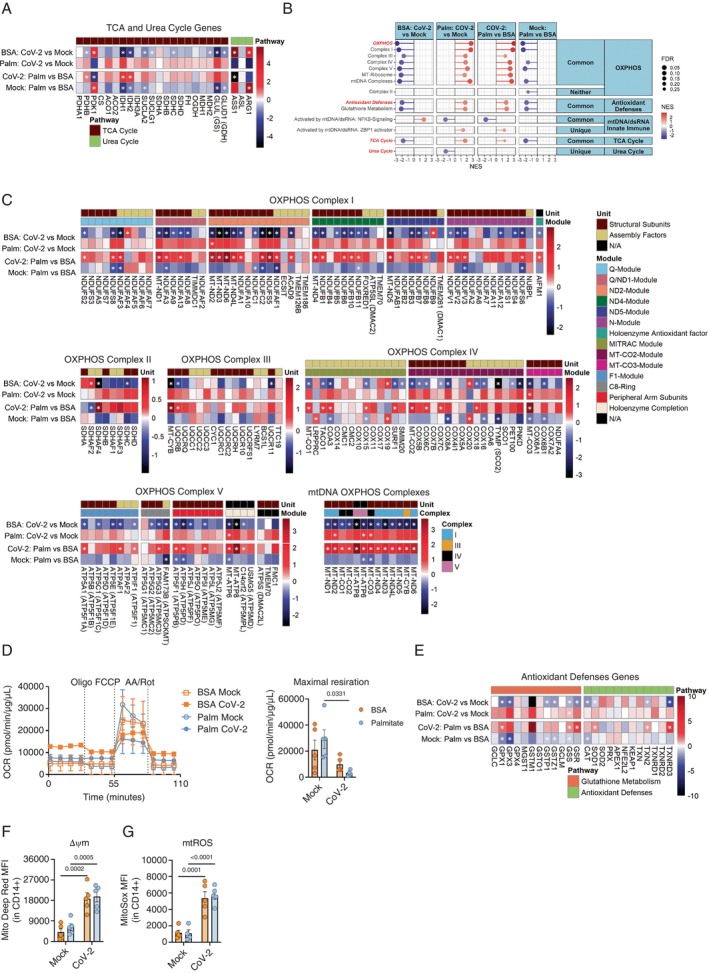
Palmitate priming enhances mitochondrial dysfunction in SARS‐CoV‐2‐infected monocytes. (A, C and E) Heatmaps of Log_2_ fold‐change values for OXPHOS, mtDNA complexes, TCA cycle and antioxidant‐related pathways from the RNA‐seq on monocytes from healthy buffy coats primed with BSA/palmitate (0.2 mM) and infected with mock or CoV‐2 (SARS‐CoV‐2, B1 variant). The colour bar represents the Log_2_ fold change values and the * indicated genes with adj. *p* value (FDR) < 0.05. (*n* = 3/group). (B) Lollipop plots for statistically significant (FDR < 0.25) up/down‐regulated pathways for custom OXPHOS, antioxidant defences, mtDNA/dsDNA complexes, TCA cycle and urea cycle pathways determined by Gene Set Enrichment Analysis (GSEA) from the RNA‐seq on monocytes from healthy buffy coats primed with BSA/palmitate (0.2 mM) and infected with mock or CoV‐2 (SARS‐CoV‐2, B1 variant). The normalised enrichment score (NES) represents the relative degree to a gene set is changed and is corrected for gene set size. The red intensity in the plot indicates the degree of upregulated pathways, and the blue intensity indicates the degree of downregulated pathways. The pathways are organised by the following: ‘Common’ = pathways are significantly regulated for both BSA and Palm CoV‐2 vs. Mock, ‘Unique’ = pathways are only significantly regulated for either BSA and Palm CoV‐2 versus Mock, and ‘Neither’ = pathways are not significantly regulated for either BSA or Palm CoV‐2 versus Mock. (*n* = 3 samples/group). (D) Monocytes were primed with BSA/palmitate for 18 h and infected with mock or CoV‐2 (SARS‐CoV‐2, B1 variant) for 1 h. Cells were washed, and 24 h later we submitted to a Mito stress test using Seahorse, and maximal respiration was determined. Seahorse was normalised by the protein content. (F and G) Monocytes primed with BSA/palmitate (0.2 mM) and infected with mock or CoV‐2 (SARS‐CoV‐2, B1 variant). The cells were washed with PBS and 24 h later stained with Mitotracker Deep Red and MitoSOX to be analysed by flow cytometry. Data represents mean ± SEM of at least three independent experiments performed in triplicate for each sample (*n* = 5 samples); two‐way ANOVA with Tukey's post hoc tests.

SARS‐CoV‐2 has deleterious effects on OXPHOS by elevating the mitochondrial membrane potential (ΔΨm) and generating mitochondrial reactive oxygen species (mtROS), which lead to the accumulation of NADH molecules [18, 19]. We observed the ablation of pathways involved in OXPHOS, including OXPHOS assembly factors, OXPHOS subunits, mtDNA complexes, the TCA cycle and mitochondrial central dogma, as shown by bulk RNA‐seq (Figure 4A–C; Supporting Information: Figure 3C,D). The mitochondrial central dogma encompasses the processes of mitochondrial gene expression, including transcription by mitochondrial RNA polymerase, RNA processing and translation by mitoribosomes, all of which are essential for maintaining mitochondrial function and energy production. Additionally, SARS‐CoV‐2 infection decreased the maximal respiratory capacity of monocytes and downregulated several genes related to glutathione metabolism and antioxidant defences (Figure 4D,E). Furthermore, SARS‐CoV‐2 infection increased ΔΨm and promoted mtROS production (Figure 4F,G, Supporting Information: Figure 4A,B). Altogether, these data indicate that SARS‐CoV‐2 infection disrupts mitochondrial metabolism in monocytes.

Extended exposure to palmitate can also impair FAO and promote oxidative stress [32]. Palmitate treatment decreased the gene expression of genes involved in the assembly and function of OXPHOS complexes and genes related to the TCA cycle (Figure 4A; Supporting Information: Figure 3C), though to a lesser extent than SARS‐CoV‐2 infection (Figure 4B,C). Palmitate‐treated monocytes displayed decreased expression of genes related to antioxidant metabolism (GPX1, GPX3, GSTP1, GSTZ1, GSS and CAT) with no changes in respiratory capacity, ΔΨm or mtROS (Figure 4D–G and Supporting Information: Figure 4A,B). Additionally, Palm+CoV‐2 induced the expression of mitophagy‐related genes and death factors (Supporting Information: Figure 3D), suggesting that prolonged exposure to palmitate has cytotoxic effects on monocytes. Further studies are needed to confirm these effects.

Considering the independent deleterious effects of SARS‐CoV‐2 and palmitate, we speculated that their combination, Palm+CoV‐2, could completely abrogate respiratory capacity. Indeed, we demonstrated that palmitate priming abolished maximal respiratory capacity, increased ΔΨm and elevated mtROS compared to Palm+Mock control (Figure 4D,F,G). Palm+CoV‐2 monocytes showed a coordinated upregulation of OXPHOS, OXPHOS assembly factors and ETC subunits (Figure 4B,C), consistent with a compensatory effort to offset the disruption of mitochondrial respiration. Similar results were observed in SARS‐CoV‐2‐infected patients, where the virus upregulates mitochondrial genes in some organs but impairs bioenergetics in others, such as the heart [19].

### 3.5 Metabolic Priming Promotes Citrate Export for Histone Acetylation in Monocytes Infected With SARS‐CoV‐2

**FIGURE 5:**
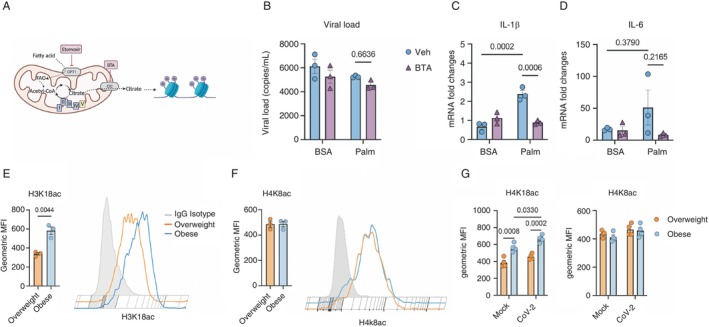
The metabolic priming of monocytes infected with SARS‐CoV‐2 depends on citrate export and histone acetylation. (A) Schematic representation of histone acetylation from fatty acid oxidation‐derived citrate. Created with Biorender. (B–D) Monocytes from healthy buffy coats were pretreated with 500 μM of BTA for 1 h, primed with BSA/palmitate (0.2 mM) for 18 h, and infected with CoV‐2 (SARS‐CoV‐2, B1 variant) for 1 h. Cells were washed, and the viral load and cytokines were measured by qPCR 24 h later. Data represent mean ± SEM of at least three independent experiments performed in triplicate for each sample (*n* = 3 samples). Two‐way ANOVA with Tukey's post hoc tests. (E–G) Blood from lean/overweight and patients living with obesity was collected, and the levels of H3K18ac and H4K8ac in CD14^+^ monocytes were analysed by flow cytometry. Data represents mean ± SEM of at least three independent experiments performed in triplicate for each sample (*n* = 3/4 samples). T‐tests or Two‐way ANOVA with Tukey's post hoc tests.

Given that the induction of the transcription machinery for OXPHOS did not result in increased respiration and that FFAs are not used as a fuel source in palmitate‐primed monocytes, we hypothesised that part of the citrate generated in FAO/β‐oxidation is exported to the cytoplasm for other cellular processes, such as FA synthesis or histone acetylation, as previously demonstrated [33]. Palmitate priming increased the expression of the mitochondrial citrate/isocitrate carrier Slc25a1, which exports citrate from the mitochondria to the cytoplasm (Figure 3B). Inhibition of Slc25a1 with the benzene‐tricarboxylate analog (BTA) (Figure 5A) was sufficient to decrease the gene expression of pro‐IL‐1β without affecting viral load and IL‐6 (Figure 5B–D and Supporting Information: Figure 4C). Thus, the exacerbated inflammation caused by palmitate priming through H3K18 acetylation in SARS‐CoV‐2‐infected monocytes relies on FAO‐derived citrate and its transport from mitochondria to the cytosol.

We next analysed the impact of palmitate on two acetylation sites, histone 3 lysine 18 (H3K18) and histone 4 lysine 8 (H4K8), by flow cytometry. Acetylation of these histone sites is associated with active transcription of genes, including those involved in regulating inflammatory cytokines, such as TNF‐α and IL‐6 [34]. H3K18 is involved in the priming effect of palmitate on IL‐6 in LPS‐activated human monocytes, suggesting a potential link between these FFAs and the priming of monocytes [35]. Additionally, it has been described that obesity elevates the acetylation of H3K9 and H3K18 lysine residues in TNF gene [36]. Incubation of monocytes with palmitate increased acetylated H3K18 but did not affect H4K8 acetylation (Supporting Information: Figure 4D,E). To understand the translational relevance of these findings, we assessed histone acetylation levels in circulating monocytes from lean/overweight individuals and individuals with obesity. In the absence of infection, monocytes from individuals with obesity showed a significant increase in acetylated H3K18, independently of SARS‐CoV‐2 infection, compared to monocytes from lean/overweight individuals (Figure 5E,F). Infection of monocytes isolated from patients with obesity exacerbated H3K18, but not H4K8, acetylation (Figure 5G). Palmitate priming of monocytes also increased H3K18 acetylation (Supporting Information: Figure 4D). Together, these findings suggest that palmitate treatment or obesity may enhance monocyte H3K18 acetylation along with increased gene expression of inflammatory cytokines, and this effect can be mimicked by palmitate treatment. We next performed H3K18 acetylation‐ChIP followed by qPCR analysis of the IL‐1β and IL‐6 promoters. We observed a trend indicating that palmitate‐primed monocytes exhibit elevated H3K18 acetylation at the IL‐6 promoter site, although this did not reach statistical significance. There was no apparent effect on IL‐1β (Supporting Information: Figure 4F). Altogether, these data suggest that obesity may induce a specific type of primed immunity mediated by palmitate, which enhances the inflammatory profile of monocytes infected with SARS‐CoV‐2. While our results show a trend in the modulation of H3K18 acetylation at the IL‐6 promoter, further studies are necessary to evaluate the broader effects of palmitate priming on different histones and cytokines to fully understand its role in modulating immune responses.

**FIGURE 6:**
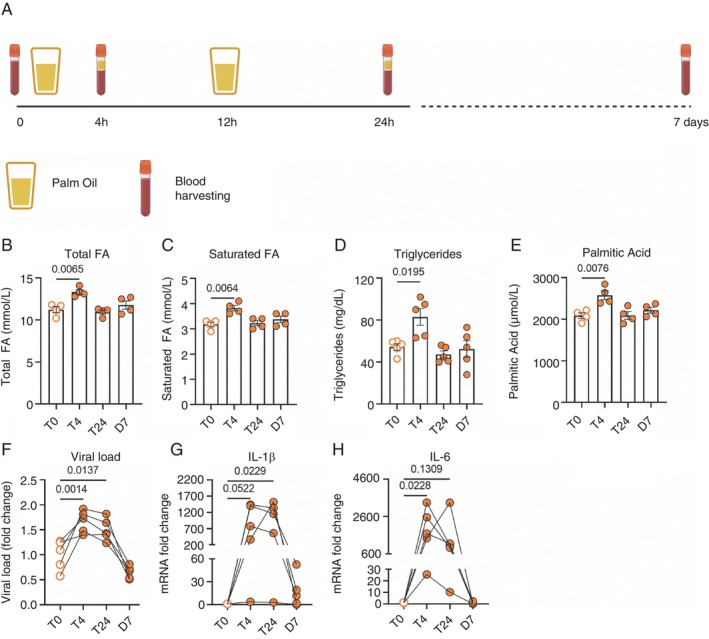
Increased levels of FFA in the circulation induce elevated susceptibility of monocytes to SARS‐CoV‐2 infection and increase their inflammatory profile. (A) Experimental design. Twelve‐h fast volunteers were admitted to the clinic, and the first blood sample was harvested (T0). After, the individuals ingested 70 mL of palm oil (50% saturated fatty acids, with 44% palmitic acid (C16:0) and 5% stearic acid (C18:0). They had their blood harvested for 4 after palm oil ingestion (T4). A second dose of palm oil was administered 12 h after the first dose. Blood samples were harvested 24 h (T24) and 7 days (D7) in overnight fasted individuals after the first administration. (B–E) Blood measurements of total fatty acids, saturated fatty acids, triglycerides and palmitic acid immediately before, 4 h, 24 h and 7 days after ingesting 150 mL of palm oil. (F–H) Monocytes from the volunteers were infected with CoV‐2 (SARS‐CoV‐2, B1 variant) for 1 h, washed with PBS, and cultured with RPMI for another 24 h. The viral load and IL‐6 levels were measured by qPCR. Data represented as mean + SEM of at least two independent experiments. Data represents mean ± SEM; One‐way ANOVA with Tukey's post hoc tests. *n* = 4 samples.

To further validate our metabolic‐primed immunity hypothesis, we tested whether increased FFA levels could induce these changes in monocytes of healthy, lean individuals (Supporting Information: Table 2). To decrease circulating FA levels, individuals underwent nutritional evaluation and dietary orientation with a nutritionist 1 week before palmitate ingestion. After a 12‐h fast, volunteers were admitted to the clinic, and the first blood sample was collected (T0). To increase circulating FA levels, the individuals ingested 70 mL of palm oil (50% saturated FA, with 44% palmitic acid [C16:0] and 5% stearic acid [C18:0]). Blood samples were collected 4 h after palm oil ingestion (T4). A second dose of palm oil was administered 12 h after the first dose, and blood samples were collected 24 h (T24) and 7 days (D7) after the first palm oil ingestion from overnight‐fasted individuals (Figure 6A).

Treatment with palm oil significantly increased circulating FA, saturated FA, triglycerides and palmitate 4 h after ingestion, which normalised to baseline levels after 24 h and 7 days (Figure 6B–E). Palm oil‐treated individuals had their circulating monocytes isolated and infected with SARS‐CoV‐2. Metabolic priming of monocytes resulted in increased viral load 4 and 24 h after palm oil ingestion (Figure 6F). Additionally, the gene expression of pro‐IL‐1β and IL‐6 was elevated at 4 and 24 h after palm oil ingestion (Figure 6G,H). Consistent with the turnover rate of monocytes in circulation, which typically lasts no longer than 7 days [37], 1 week after monocyte priming, viral load and gene expression of pro‐IL‐1β and IL‐6 returned to baseline (Figure 6F–H), suggesting that the metabolic reprogramming were not epigenetically imprinted in haematopoietic progenitors. Further studies are needed to assess whether immune cells primed by metabolic factors can respond to a secondary challenge. Altogether, these results demonstrate that increased levels of FFAs in circulation can elevate monocyte susceptibility to infection in short periods of time and promote an inflammatory profile, supporting the concept of metabolic‐primed immunity.

## 4 Discussion

We previously showed that SARS‐CoV‐2‐infected monocytes exhibit dysfunction in OXPHOS, leading to increased mtROS production, HIF‐1α‐induced glycolysis and subsequently enhanced expression of inflammatory cytokines (cytokine storm) [18, 38]. We showed that the cytokine storm caused lung epithelial cell death, T‐cell dysfunction and exhaustion [18, 38]. Here, we demonstrate that monocytes from nondiabetic individuals living with obesity are more susceptible to SARS‐CoV‐2 infection, which results in higher gene expression of inflammatory cytokines. We also observed that priming monocytes with palmitate in vitro replicates the increased viral load and cytokine gene expression seen in monocytes isolated from non‐diabetic individuals with obesity infected with SARS‐CoV‐2. Our data suggest that exposure of monocytes to FA promotes epigenetic modifications that enhance viral load and pro‐inflammatory responses upon SARS‐CoV‐2 infection.

Among immune cells, 6% of the circulating monocytes from COVID‐19‐infected patients are infected with SARS‐CoV‐2 [39]. In response to pathogens or damaged molecules, innate immune cells, including monocytes and macrophages, rewire their metabolism to a glycolytic state [40]. SARS‐CoV‐2 infection or palmitate treatment of monocytes activates a proinflammatory program associated with HIF‐1α stabilisation and increased glycolytic metabolism [18, 28, 41]. Our RNA‐seq data suggest that palmitate‐primed monocytes infected with SARS‐CoV‐2 enhanced FA metabolism, as indicated by the decreased viral load in etomoxir‐treated cells. SARS‐CoV‐2 utilises host lipid‐related metabolic pathways for replication [33], including lipid storage compartments such as lipid droplets, which serve as replicative centres [42]. SARS‐CoV‐2‐infected monocytes depend on lipid droplets for their proinflammatory activation [33]. Since obesity increases lipid content in macrophages [43], the elevated levels of FA within monocytes during obesity can create a favourable environment for viral replication that is independent of glucose metabolism.

In line with literature suggesting that SARS‐CoV‐2 downregulates mitochondrial machinery [19, 44], we show that SARS‐CoV‐2 infection abolishes mitochondrial respiration and the transcription of genes related to ETC complexes [19]. Due to host–viral protein interactions with mitochondrial proteins, OXPHOS may remain inhibited if the virus is present, regardless of mitochondrial transcript levels. Our data suggest that SARS‐CoV‐2 infection‐mediated inhibition of OXPHOS may disrupt the TCA cycle, accumulating succinate and citrate. Similar to LPS‐activated macrophages, which enhance metabolite flux through glycolysis and the TCA cycle while channelling carbons for acetyl‐CoA‐dependent histone acetylation via ATP citrate lyase [34], our data suggest that palmitate‐primed monocytes rely on citrate export from mitochondria to promote histone acetylation. However, our limited results regarding the viral load in monocytes treated with the citrate transporter inhibitor suggest that palmitoyl‐CoA formation in the cytosol might serve as an independent source of acetyl‐CoA units for histone acetylation, as demonstrated in the THP‐1 human monocyte cell line [35]. Further studies using C13‐labelled metabolites are needed to address these points fully.

Acetylation of H3K18 has been described as involved in the priming effect of palmitate on IL‐6 expression in LPS‐activated human monocytes [35]. Additionally, obesity increases the acetylation of H3K9 and H3K18 lysine in the TNF promoter [36]. IL‐6 is an important therapeutic target, and IL‐6 blockers have been repurposed to treat cytokine storms in severe COVID‐19 patients [45], highlighting IL‐6 as a key pro‐inflammatory cytokine in the pathophysiology of COVID‐19 and its severe cases. Our findings suggest that metabolic reprogramming influences IL‐6 expression. However, our results do not fully elucidate the effects of the acetylation of the IL‐6 promoter, and further studies are needed to explore this in more detail. This suggests a potential mechanism for the elevated levels of IL‐6 commonly observed in severe COVID‐19 patients, particularly people living with obesity. Finally, as proof of concept, we demonstrated that transient elevation of FFAs in the circulation of lean and healthy volunteers can induce a strong secondary inflammatory response in monocytes infected with SARS‐CoV‐2.

Calorie restriction has been widely associated with increased lifespan [46]. Additionally, it has been shown to enhance insulin sensitivity in individuals with obesity [47]. Emerging evidence suggests that calorie restriction benefits immune function by improving T cell responses and maintaining thymic function [48]. Based on these findings, we speculate that calorie restriction may lower circulating FFA levels, thereby modulating the inflammatory response to SARS‐CoV‐2 infection in a beneficial manner, which should be further investigated.

In conclusion, our study provides evidence that elevated FA, commonly found in individuals living with obesity, induce a specific type of primed immunity, which we term metabolic primed immunity. This process is triggered by the acetylation of histones in the promoters of specific cytokines. The resulting epigenetic changes lead to a dysregulated and exacerbated inflammatory profile in monocytes upon SARS‐CoV‐2 infection, contributing to cytokine storms associated with severe acute respiratory syndrome. Managing FFA levels in individuals highlights potential therapeutic targets that could be used to downregulate inflammation and improve clinical outcomes in severe patients.

Moreover, our findings may have broader implications beyond SARS‐CoV‐2. For instance, other viruses that infect monocytes, such as cytomegalovirus (CMV) and HIV, could also exploit metabolic‐primed immunity mechanisms. While our study focuses on SARS‐CoV‐2, the concept of metabolic‐primed immunity may be relevant to understanding the inflammatory responses in infections with these viruses. CMV and HIV are known to affect monocyte function and contribute to chronic inflammation and immune dysregulation. Future studies should investigate whether similar metabolic reprogramming and histone acetylation processes occur in monocytes during CMV and HIV infections, and how these mechanisms might impact disease severity and progression.

## 5 Limitations of the Study

This study has several limitations. One limitation of our study is the relatively small sample size, which may limit the generalisability and statistical power of the findings. Additionally, while we observed changes in gene expression for key cytokines such as pro‐IL‐1β and IL‐6, we did not measure these cytokines at the protein level, which would provide a more comprehensive understanding of the inflammatory response. Furthermore, the study design primarily focuses on the effects of priming without considering the potential impact of continuous exposure to palmitate. This distinction could affect the interpretation of the observed metabolic reprogramming and its relevance to long‐term immune responses. These limitations highlight the need for further studies with larger sample sizes, protein‐level measurements and alternative models better to assess the effects of metabolic reprogramming on immune function. Our findings indicate that FFA‐induced changes in monocyte metabolism and effector functions are transient and dependent on the continued presence of FFAs, without evidence of long‐term reprogramming or epigenetic modifications characteristic of trained immunity. We did not assess changes at the bone marrow level or after a washout/rest period, which are essential for establishing trained immunity. Additionally, the limited amount of blood available from the people with obesity non‐diabetic group, mainly from unvaccinated individuals and those never infected with SARS‐CoV‐2, restricted our ability to validate findings in this subgroup. Since both vaccination and prior infection can alter immune profiles, the absence of these factors limits our interpretation. Our experiments were performed ex vivo in a controlled environment, making the findings context dependent. While the inhibitors used in this study are well‐characterised with established targets, we cannot exclude the possibility of unknown off‐target effects. Moreover, we could not perform C13 metabolomics or ChIP‐seq analyses due to resource constraints. This could have provided more profound insights into the metabolic pathways and transcriptional regulation underlying the observed phenomena. Finally, while we observed metabolic reprogramming in monocytes, our study does not demonstrate sustained epigenetic modifications or long‐term memory. Thus, the changes we report should be considered transient priming rather than trained immunity. Despite these limitations, our study highlights the role of metabolic priming in monocyte function and underscores the importance of distinguishing between transient priming and sustained reprogramming in immune responses.

## Author Contributions

**Gustavo Gastão Davanzo, Bianca Gazieri Castelucci, Gabriela Fabiano de Souza and Pedro M. Moraes‐Vieira:** conceptualisation, methodology, validation, visualisation, writing – original draft preparation and writing – review and editing. **Gustavo Gastão Davanzo, Bianca Gazieri Castelucci, Gabriela Fabiano de Souza, Stéfanie Primon Muraro, Larissa Menezes dos Reis, Isabella Bonilha de Oliveira, José Luís Fachi, João Victor Virgilio‐da‐Silva, Marcelo Rodrigues Berçot, Mariane Font Fernandes, Sarah de Oliveira, Nathalia Vitoria Pereira Araujo, Guilherme Ribeiro, Gisele de Castro, Webster Leonardo Guimarães Costa, Adriana Leandra Santoro, Helison Rafael P. do Carmo and Ikaro Breder:** investigation. **Pedro M. Moraes‐Vieira:** funding acquisition. **Gabriela Flavia Rodrigues‐Luiz and Afshin Beheshti:** data curation, software. **Marcelo A. Mori, Alessandro S. Farias, Daniel Martins‐de‐Souza, Joseph W. Guarnieri, Douglas C. Wallace, Patrick Varga‐Weisz, Marco Aurélio Ramirez Vinolo, José Luiz Proença‐Módena, Afshin Beheshti, Andrei C. Sposito and Pedro M. Moraes‐Vieira:** provided resources, reviewed and edited the manuscript. **Pedro M. Moraes‐Vieira:** supervised the study.

## Conflicts of Interest

The authors declare no conflicts of interest.

## Supporting information


**Data S1.** Supporting Information.

## Data Availability

The data that support the findings of this study are available from the corresponding author upon reasonable request.
