# Supplementary material for: Obesity‐Induced Metabolic Priming Exacerbates SARS‐CoV‐2 Inflammation
Source: Immunology. 2025 Apr 23;175(3):323–38. doi: 10.1111/imm.13934 (PMC12130675; doi:10.1111/imm.13934)
Supplement: Supplementary file 1 — Data S1. Supporting Information. [file IMM-175-323-s001.pdf]

Supplementary Material

Obesity-induced metabolic priming exacerbates SARS-CoV-2 inflammation.

Gustavo Gastão Davanzo[1,†], Bianca Gazieri Castelucci[1,†], Gabriela Fabiano de Souza[2,†], Stéfanie Primon Muraro[2], Larissa Menezes dos Reis[1,] Isabella Bonilha de Oliveira[4], José Luís Fachi[3], João Victor Virgilio-da-Silva[1], Marcelo Rodrigues Berçot[1], Mariane Font Fernandes[3], Sarah de Oliveira[3], Nathalia Vitoria Pereira Araujo[3], Guilherme Ribeiro[1], Gisele de Castro[1], Webster Leonardo G Costa[1], Adriana Leandra Santoro[7], Gabriela Flavia Rodrigues-Luiz[1], Helison Rafael P. do Carmo[4], Ikaro Breder[4], Marcelo A. Mori[8,9,12], Alessandro S. Farias[6,9], Daniel Martins-de-Souza[7,9,12], Joseph W. Guarnieri[5], Douglas C. Wallace[5], Marco Aurélio Ramirez Vinolo[3,9], José Luiz Proença-Módena[2,9], Afshin Beheshti[10,11], Andrei C Sposito[3,#], Pedro Manoel M. Moraes-Vieira[1,8,9,#,*].

[1]Laboratory of Immunometabolism, Department of Genetics, Evolution, Microbiology and Immunology, Institute of Biology, University of Campinas, SP, Brazil.

[2] Laboratory of Emerging Viruses, Department of Genetics, Evolution, Microbiology and Immunology, Institute of Biology, University of Campinas, SP, Brazil.

[3]Laboratory of Immunoinflammation, Department of Genetics, Evolution, Microbiology, and Immunology, Institute of Biology, University of Campinas, Campinas, SP, Brazil

[4]Department of Clinical Medicine, School of Medical Sciences, University of Campinas, Campinas, SP, Brazil.

[5]Center for Mitochondrial and Epigenomic Medicine, Division of Human Genetics, The Children's Hospital of Philadelphia, Philadelphia, PA 19104, USA.

[6]Autoimmune Research Laboratory, Department of Genetics, Microbiology, and Immunology, Institute of Biology, University of Campinas (UNICAMP), Campinas, Brazil.

[7]Laboratory of Neuroproteomics, Department of Biochemistry and Tissue Biology, Institute of Biology, University of Campinas (UNICAMP), Campinas, Brazil.

[8]Obesity and Comorbidities Research Center (OCRC), University of Campinas, SP, Brazil.

[9]Experimental Medicine Research Cluster (EMRC), University of Campinas, SP, Brazil.

[10]Broad Institute of MIT and Harvard, Cambridge, MA, USA

[11]Center for Space Biomedicine, McGowan Institute for Regenerative Medicine, Department of Surgery, University of Pittsburgh, Pittsburgh, PA, 15219, USA

[12]Department of Biochemistry and Tissue Biology, Institute of Biology, University of Campinas (UNICAMP), Campinas, SP, Brazil

[†]These authors contributed equally

[#]Co-senior authors

*Corresponding Author and Lead Contact: Prof. Pedro Manoel Mendes de Moraes Vieira. Rua Monteiro Lobato, 255, Bl H, Instituto de Biologia, Universidade de Campinas, Campinas, Brazil, 13083-862. E-mail: pmvieira@unicamp.br

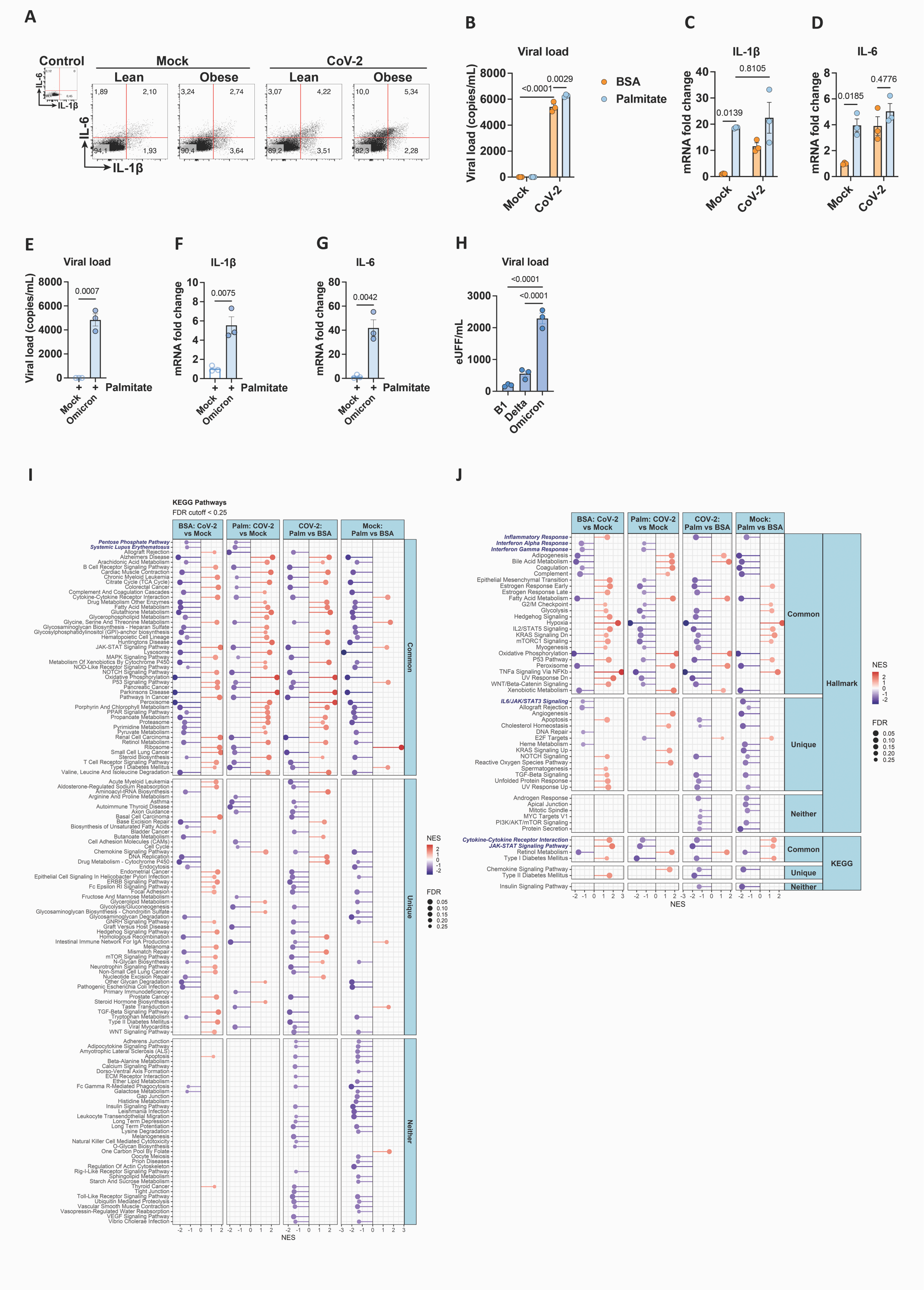

Supplementary Figure 1. Palmitate priming aggravates the inflammatory profile of monocytes infected with SARS-CoV-2. **A** – Representative flow cytometry of gated CD45^{+}CD14^{+} monocytes. First, cells were selected based on size (FSC) and granularity (SSC); second, duplets were removed and live cells gated; CD45^{+}CD14^{+} cells were defined as monocytes, as shown in Supplementary Figure 4C. Control refers to IL-6 and IL-1β FMO control. **B - D** -

Monocytes from healthy buffy coats were primed with BSA/palmitate (0.2mM) for 18h and infected with CoV-2 (SARS-CoV-2, B1 variant) for one h. Cells were washed with sterile PBS, and the viral load and cytokines were measured by qPCR 24h later. Data represents mean ± SEM of at least three independent experiments performed in triplicate for each sample (n = 3 samples); two-way ANOVA with Tukey's post hoc tests. **E - G** - Monocytes from healthy buffy coats were primed with palmitate for 18h and infected with the omicron variant of SARS-CoV-2 for one h. Cells were washed, and the viral load and cytokines were measured by qPCR 24h later. Data represents mean ± SEM of at least three independent experiments performed in triplicate for each sample: n = 3 samples. T-test. **H** - Monocytes that were pre-treated with palmitate for 24 hours. Following this, the monocytes were washed with PBS and subsequently infected with three variants of SARS-CoV-2 – B1, delta, and omicron – for 1 hour. After the infection, the virus was removed, and the cells were washed with PBS before being supplied with a fresh RPMI medium. RNA extraction was performed 24 hours later, and the viral load was quantified. Data represents mean ± SEM of at least three independent experiments performed in triplicate for each sample (n = 3 samples); One-way ANOVA with Tukey's post hoc tests. **I - J** - Lollipop plots were generated to display statistically significant (FDR < 0.25) KEGG pathways identified by Gene Set Enrichment Analysis (GSEA) using RNAseq data from monocytes isolated from healthy buffy coats. These monocytes were primed with BSA/palmitate (0.2mM) for 18 hours and subsequently infected with CoV-2 (SARS-CoV-2, B.1 variant). The normalized enrichment score (NES) indicates the relative degree of change in gene sets and is adjusted for gene set size. In the plots, red intensity signifies the degree of upregulated pathways, while blue intensity signifies the degree of downregulated pathways. Pathways are categorized as 'Common' if they are significantly regulated in both BSA and Palmitate CoV-2 vs. Mock conditions, 'Unique' if they are only significantly regulated in either BSA or Palmitate CoV-2 vs. Mock conditions, and 'Neither' if they are not regulated considerably in either BSA or Palmitate CoV-2 vs. Mock conditions. Each group consists of n = 3 samples/group.

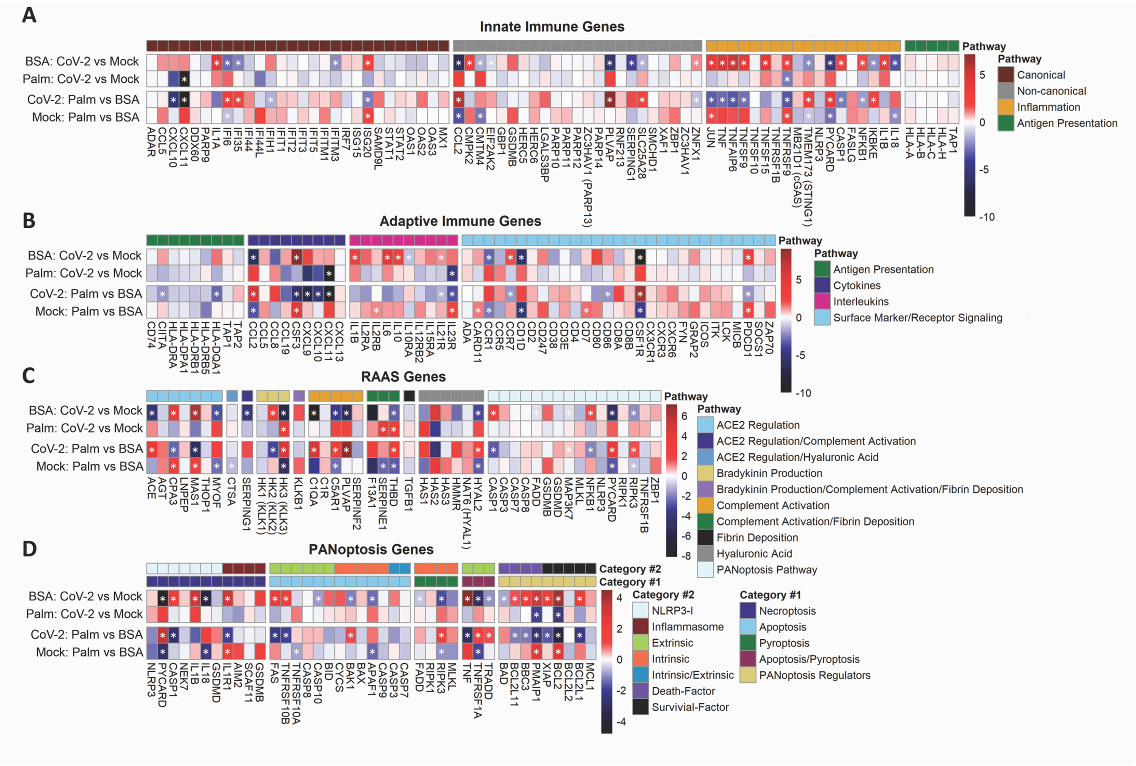

Supplementary Figure 2. Profile of immune-system-related pathways in BSA/Palmitate primed monocytes infected or not with SARS-CoV-2. A-D - Heatmaps of Log_2 fold-change values for custom Innate Immune, Adaptive Immune, RASS, and PANoptosis pathways for RNAseq data from monocytes from healthy buffy coats were primed with BSA/palmitate (0.2mM) for 18h and infected with CoV-2 (SARS-CoV-2, B1 variant). The color bar represents the Log_2 fold change values and the * indicated genes with adj. p-value (FDR) < 0.05. (N=3/group).

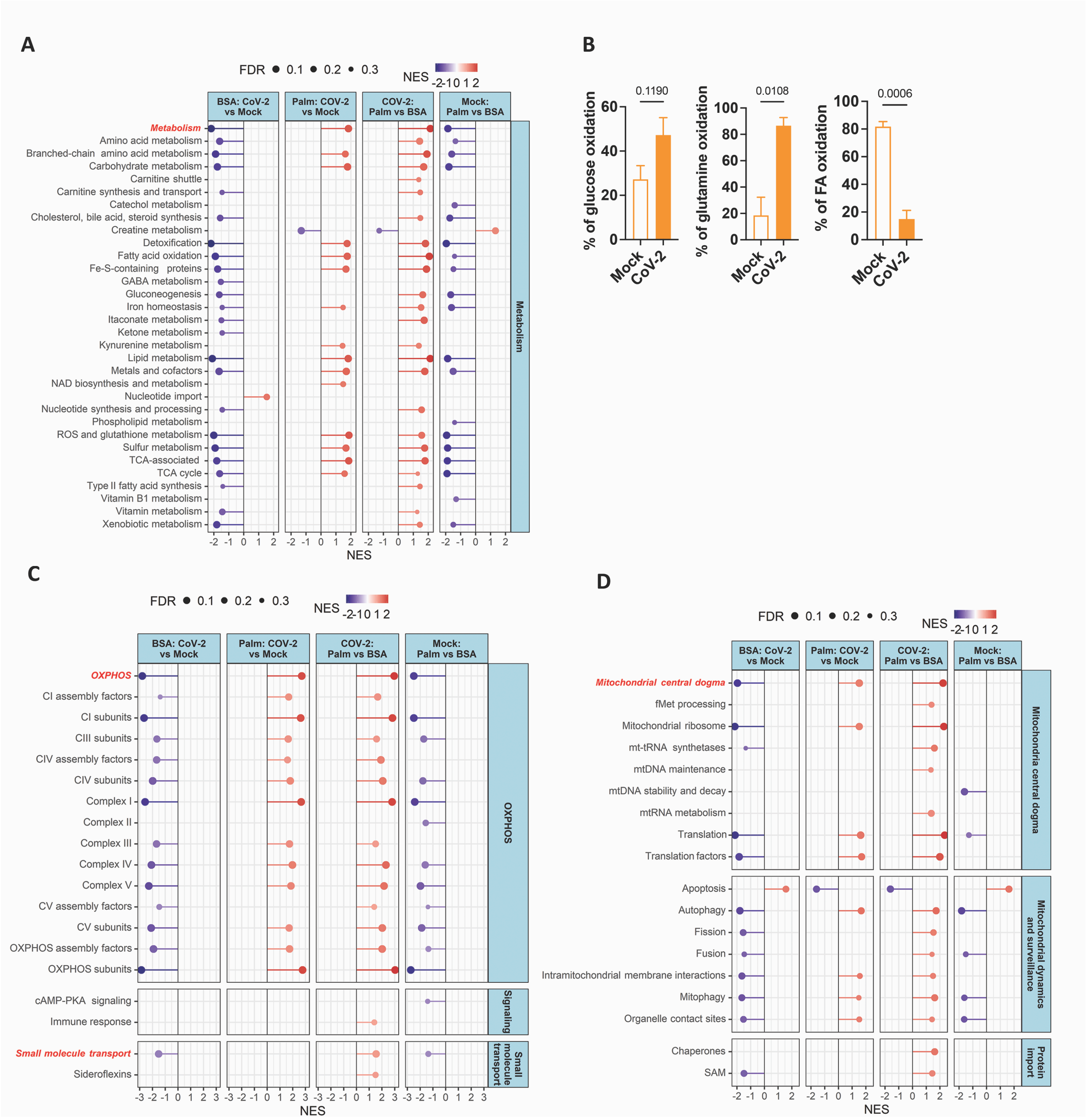

Supplementary Figure 3. Profile of mitochondrial pathways in BSA/Palmitate primed monocytes infected or not with SARS-CoV-2. A, C, and D - Lollipop plots for statistically significant mitochondrial pathways (FDR cutoff < 0.25) determined by Mito Pathways 3.0. NES, normalized enrichment score from the RNA-seq on monocytes from healthy buffy coats primed with BSA/palmitate (0.2mM) and infected with mock or CoV-2 (SARS-CoV-2, B1 variant). (n = 3 samples/group). **B** - Monocytes from healthy buffy coats were infected with CoV-2 (SARS-CoV-2 variant B1) for one h. Cells were washed with PBS, and fresh RPMI was replenished. The oxidation of glucose, glutamine, and FA was measured using a Seahorse Mito Fuel Flex test kit. Data represents mean ± SEM of at least three independent experiments performed in triplicate for each sample. n = 5 sample. T-test.

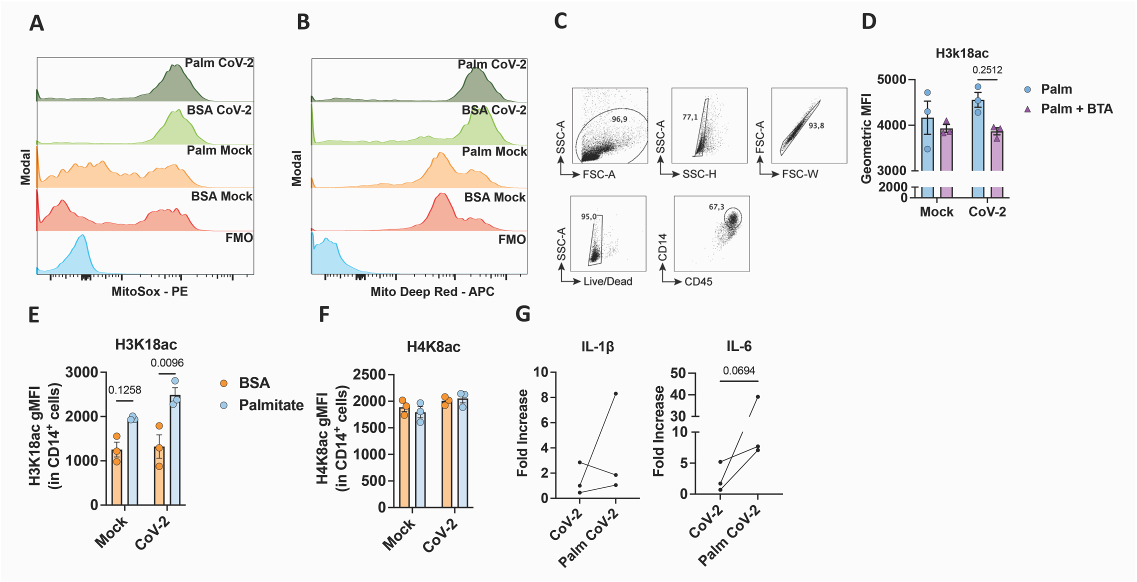

Supplementary Figure 4. Histone acetylation is associated with SARS-CoV2-infected monocytes' inflammatory profile. A – B Representative histograms of gated CD45$^+$CD14$^+$ monocytes stained with MitoSox (mitochondria reactive oxygen species) and Mito Deep Red (mitochondria membrane potential). **C** – Gate strategy of monocyte isolation for flow cytometry, all flow cytometry data was gated as shown here. First, cells were selected based on size (FSC) and granularity (SSC); second, duplets were removed; live cells were gated; CD45$^+$CD14$^+$ cells were defined as monocytes. **D** - Monocytes from healthy buffy coats were pretreated with 500µM of BTA for 1hr, primed with BSA/palmitate (0.2mM) for 18hrs, and infected with CoV-2 (SARS-CoV-2, B1 variant) for one hour. The levels of H3K18ac in CD14$^+$ monocytes were analyzed by flow cytometry. Data represents mean ± SEM of at least three independent experiments performed in triplicate for each sample (n = 3 samples); two-way ANOVA with Tukey's post hoc tests. **E - F** – H3K18 and H4K8 acetylation in palmitate-treated and SARS-CoV-2 infected monocytes. Data represents mean ± SEM of at least three independent experiments performed in triplicate for each sample (n = 3 samples); two-way ANOVA with Tukey's post hoc tests. **G** - H3K18ac chromatin immunoprecipitation qPCR was performed on BSA/Palmitate-primed monocytes infected with CoV-2 (SARS-CoV-2, B.1 variant). n = 3 samples; Paired T test.

Supplementary Table 1 – Baseline characteristics of participants

	Overweight	Obese	*p-value*
**n**	9	5	
**Demographics**			
Age. years	45±7	42±12	
Male. %	78	80	
**Medical history**			
Time with diabetes. years	-	-	
BMI. Kg/m^2	26±3	33±3	*0.0015*
Hypertension. %	0	40	
Distal diabetic neuropathy. %	-	-	
HOMA-IR	2.5±1.7	2.6±1.6	*0.9130*
**Blood pressure**			
Systolic blood pressure. mmHg	120±6	138±8	*0.0005*
Diastolic blood pressure. mmHg	77±5	88±6	*0.0040*
**Biochemical analysis**			
Fasting blood glucose. mg/dL	98±6	97±6	*0.7959*
Insulin. mUI/mL	10±7	11±6	*0.9031*
A1c. %	5.0±0.3	5.5±0.3	*0.0178*
Total cholesterol. mg/dL	199±27	188±41	*0.5389*
LDL-C. mg/dL	124±22	124±29	*0.9963*
HDL-C. mg/dL	49±10	40±11	*0.1326*
VLDL-C. mg/dL	26±12	24±7	*0.7392*
Triglycerides. mg/dL	142±106	124±53	*0.7184*
Creatinine. mg/dL	0.94±0.13	0.93±0.11	*0.8500*

Supplementary Table 2 – Fatty acid measurements in the blood of the patients who participated in the palm oil ingestion protocol.

General				
Age. years	30.3±3.3			
BMI. Kg/m^2	22.8±0.9			
N	4			
	**T0**	**T4**	**T24**	**D7**
Myristic acid, C14:0 (umol/L)	104.0±16.0	105.7±14.3	125.5±35.3	104.2±13.1
Palmitoleic acid, C16:1w7 (umol/L)	183.0±39.3	192.7±46.8	103.5±7.4*	198.2±38.6
Palmitic acid, C16:0 (umol/L)	2084.5±137.6	2575.5±209.0*	2090.5±184.5	2216.2±144.1
γ-Linolenic acid, C18:3w6 (umol/L)	43.5±7.5	48.5±15.9	35.8±8.2	51.5±27.5
α-Linolenic acid, C18:3w3 (umol/L)	67.3±14.3	107.5±19.7*	64.8±2.9	64.3±11.4
Linolenic acid, C18:2w6 (umol/L)	3467.5±179.8	3972.8±92.9*	3693.8±150.5	3556.8±357.8
Oleic acid, C18:1w9 (umol/L)	1523.8±53.1	2104.3±214.0*	1497.8±102.5	1645.3±147.3
Vaccenic acid, C18:1w7 (umol/L)	281.0±57.3	334.8±23.9	220.5±52.4	305.3±31.5
Stearic acid, C18:0 (umol/L)	821.0±54.0	977.3±54.4	804.5±86.8	889.3±95.6
Arachidonic acid, C20:4w6 (umol/L)	1606.0±213.6	1765.5±118.1	1405.5±118.0	1644.0±196.7
Docosahexaenoic acid DHA, C22:6w3 (umol/L)	279.0±130.0	304.0±122.9	210.8±81.5	297.3±125.1
Docosapentaenoic acid DPA, C22:5w6 (umol/L)	50.3±15.8	56.8±14.2	41.8±9.7	51.5±4.9
Docosapentaenoic acid DPA, C22:5w3 (umol/L)	103.8±11.0	120.0±19.4	91.8±15.5	114.8±27.1
Docosatetraenoic acid DTA, C22:4w6 (umol/L)	61.8±5.6	70.0±10.1	54.8±5.9	66.5±16.0
Docosahexaenoic acid, C22:1 (umol/L)	5.5±1.3	6.3±0.5	5.5±1.0	5.5±0.6
Nervonic acid, C24:1w9 (umol/L)	71.8±7.4	75.8±4.9	71.0±7.0	71.5±8.7
Total saturated fatty acids(mmol/L)	3.2±0.2	3.8±0.2*	3.2±0.2	3.4±0.3
Total monounsaturated fatty acids (mmol/L)	2.1±0.2	2.8±0.3*	1.9±0.1	2.3±0.3
Total polyunsaturated fatty acids (mmol/L)	5.9±0.5	6.7±0.3	5.8±0.3	6.1±0.6
Total fatty acids (mmol/L)	11.2±0.7	13.3±0.6*	11.0±0.5	11.8±1.0

* $p<0.05$ vs T0 control - One-Way Anova.

Supplementary Table 3 – Reagents used in the manuscript.

REAGENT	SOURCE	IDENTIFIER
**Antibodies**		
anti-CD14-PE-Cy7 (clone M0P9)	BD Biosciences	#557831
Anti-Histone H3 (acetyl K18)	Abcam	#ab1191
Anti-Histone H4 (acetyl K8)	Abcam	#ab45166
**Chemicals**		
1,2.3-benzenetricarboxylic acid hydrate (BTA)	Sigma Aldrich	B4201
Etomoxir sodium salt	Cayman	#11969
MitoTracker Deep Red	Thermo Fisher Scientific	#M22426
MitoSox Red Mitochondrial ROS indicator	Thermo Fisher Scientific	#M36008
BD Horizon Fixable Viability Stain 510	BD Biosciences	#564406
TRIzol Reagent	Sigma Aldrich	#15596026
Paraformaldehyde	Sigma Aldrich	#F1635
Ficoll Plaque Plus	Sigma Aldrich	#GE17-1440-03
RPMI 1640	Thermo Fisher Scientific	#11875119
Penicilin-Streptomycin	Sigma Aldrich	#P4333
Fetal Bovine Serum (FBS)	Thermo Fisher Scientific	#12657029
Bovine Serum Albumine fatty acid free	Sigma Aldrich	#A7030
Sodium Palmitate	Sigma Aldrich	#P9767
GoScript™ Reverse Transcriptase cDNA	Thermo Fisher Scientific	#4311235
QuantiNova SYBR Green PCR kit	Qiagen	#208056
Seahorse XF Cell Mito Stress Kit	Agilent	#103015-100
Seahorse XF Glycolysis Stress Test Kit	Agilent	#103017-100
Seahorse XF Mito Fuel Flex Test Kit	Agilent	#103270-100
Protein A and G	Invitrogen	#10001D, #10003D
MinElute PCR purification kit	Qiagen	#28004
RNAeasy kit	Qiagen	#74104
RNAse	Invitrogen	#12091021

Supplementary Table 4 – Primers used in the manuscript.

Primers		
h18s	Forward	CCCAACTTCTTAGAGGGACAAG
	Reverse	CATCTAAGGGCATCACAGACC
hIL-1B	Forward	AAGCTGATGGCCCTAAACAG
	Reverse	AGGTGCATCGTGCACATAAG
hIL-6	Forward	AGTGAGGAACAAGCCAGAGC
	Reverse	AGCTGCGGCAGAATGAGATGA
hIL-1B promotor	Forward	AACAGCGAGGGAGAAACTGG
	Reverse	AATCCCAGAGCAGCCTGTTG
hIL-6 promotor	Forward	GCTAGCCTCAATGACGACCT
	Reverse	TGGGGCTGATTGGAAACCTT
